# Needles for healing: stem cells, platelet-enriched plasma, and fat injection in perianal fistulizing Crohn’s disease

**DOI:** 10.1007/s10151-026-03318-4

**Published:** 2026-05-13

**Authors:** E. Takata, J. J. Lee, A. L. Lightner

**Affiliations:** 1 Department of Colorectal Surgery, Scripps Clinic, 10667 N. Torrey Pines Rd, La Jolla, CA 92037 USA; 2 Department of Surgery, Naval Medical Center San Diego, San Diego, CA USA; 3 Department of Molecular and Cellular Biology, The Scripps Research Institute, La Jolla, CA USA; 4 Department of Immunology and Microbiology, The Scripps Research Institute, La Jolla, CA USA; 5 Calibr-Skaggs, The Scripps Research Institute, La Jolla, CA USA

**Keywords:** Perianal fistulas, Crohn’s disease, Mesenchymal stem cell therapy, Platelet rich plasma, Autologous fat injection

## Abstract

Perianal fistulizing Crohn’s disease affects a third of patients with Crohn’s disease and represents one of the most challenging complications to manage. This severe phenotype is characterized by aggressive disease behavior, high recurrence rates, frequent hospitalizations and surgical interventions, and profound impairment of quality of life, particularly affecting social and sexual function. The complex pathophysiology involves genetic susceptibility, immune dysregulation with elevated inflammatory cytokines, epithelial-to-mesenchymal transition, myofibroblast activation, and impaired wound healing mechanisms. Despite advances in anti-inflammatory and immunomodulatory therapies, current medical and surgical approaches achieve long-term fistula healing in only approximately 50% of patients. More than 90% of patients undergo multiple operative interventions, often with limited efficacy and risk of fecal incontinence. Anti-tumor necrosis factor agents, particularly infliximab, remain the cornerstone of medical therapy, yet more than 50% of patients lose response over time. Combination approaches with setons and biologics improve outcomes but remain suboptimal for many patients.These persistent limitations have prompted increasing interest in regenerative strategies aimed at restoring tissue integrity and enhancing local healing mechanisms. Mesenchymal stem cell therapy, particularly adipose-derived stem cells, has emerged as a promising approach, with clinical trials demonstrating complete fistula healing in the majority of cases and a favorable safety profile, but there are limitations with logistics of cell handling and negative late phase pivotal trials. This review evaluates the current landscape of novel regenerative therapies for perianal fistulizing Crohn’s disease, including mesenchymal stem cell preparations, extracellular vesicle-based approaches, and adjunctive techniques.

## Introduction

Crohn’s disease (CD) is a chronic inflammatory disease of the gastrointestinal tract exhibiting characteristic transmural inflammation with heterogeneous clinical presentations [1]. The presenting phenotype of CD varies among individuals; up to 33% of patients with Crohn’s disease will develop a perianal manifestation with perianal fistulizing disease [2, 3]. Fistulas are abnormal connections between two surfaces that are epithelial lined, and more specifically perianal fistulas exist between the mucosal layer of the anal canal and the surrounding skin, thought to occur in CD owing to the transmural involvement [4, 5]. Unfortunately, perianal fistulizing Crohn’s disease (PFCD) is aggressive and is associated with high recurrence, hospitalizations, surgeries, psychological distress, and diminished quality of life [6].

Owing to the complex pathophysiology of perianal fistulas, especially in the context of CD, and complexity in the anatomy around the anal canal and sphincter complex, perianal fistulizing disease is notoriously difficult to treat and accurately diagnose. Patients presumed to have idiopathic perianal fistulas may also have CD, but the boundaries between these diagnoses remain unclear making confident classification difficult. Isolated perianal CD is characterized by a lack of luminal inflammation resulting in exclusion from luminal IBD criteria; however, overlapping and poorly defined diagnostic criteria complicate accurate phenotype identification. This lack of clarity may result in undiagnosed or untreated CD leading to negative patient outcomes [7]. Additionally, there is extremely limited understanding of the pathogenesis of PFCD, which contributes to the extreme difficulty and low success rate in achieving true remission. As we learn more about the pathogenesis of PFCD, including a complex interaction of genetic susceptibility, host and microbial factors, and several modifiable influences including uncontrolled inflammation, impaired wound healing, epithelialized tracts, and high-pressure anatomic connections that together drive fistula development and persistence, we continue to investigate ways to improve patient outcomes [8, 9].

While clinicians agree that treatment plans require a multidisciplinary approach, finding a definitive treatment has proven to be extremely challenging. Medical treatment includes anti-inflammatory and immunomodulatory regimens that have inherent risk of potentially significant effects. Regardless of all available medical therapies, greater than 90% of patients undergo multiple surgical interventions in attempt to achieve fistula closure, all of which have increased risk of fecal incontinence [10].

Given these substantial challenges, it has become increasingly clear that conventional medical and surgical strategies often fall short in achieving durable remission for PFCD. Persistent symptoms, frequent recurrences, and the potential for treatment-related complications underscore the need for approaches that directly support tissue repair and restore function. As clinicians and researchers deepen their understanding of the biologic processes driving fistula formation, it has opened the door to therapies that move beyond inflammation control alone. This growing interest in regenerative techniques reflects a shift toward treatments designed to enhance wound healing at the cellular level. In this context, emerging options such as mesenchymal stem cell therapy, platelet-rich plasma injections, and autologous fat–based interventions are being investigated as promising avenues for improving outcomes in PFCD [11–14].

This comprehensive review examines the emerging therapeutic strategies for PFCD by exploring the novel therapies currently under investigation, summarizing key clinical trials to assess their effectiveness and limitations, and highlighting the outcomes and future directions shaping this rapidly evolving field.

## Mesenchymal stem cells in perianal fistulas

### Background

Mesenchymal stem cells (MSCs) are pluripotent cells that have the ability to differentiate into multiple different mature cell types and are thought to act through anti-inflammatory, immunomodulatory, pro-angiogenic, and tissue-repair mechanisms with low immunogenicity, enabling autologous and allogeneic approaches [15]. The application of MSC therapy has been widely investigated and utilized in several severe diseases, particularly when previously established treatment options fail, demonstrating promising results in myocardial infarction, severe autoimmune diseases such as rheumatoid arthritis, liver cirrhosis, and spinal cord injury [16]. MSCs can be isolated from a variety of tissues, including umbilical cord, bone marrow, and adipose tissue; the latter two are most frequently utilized as they are more easily sourced. Once isolated, MSCs can be administered in a variety of ways including intravenous infusion, inhalation, or local injections to the damaged site [17]. MSC therapy delivered as a single, local injection into the fistula tract has emerged as a guideline-supported option for PFCD when previously established therapies such as anti-TNF agents, seton drainage, and surgery have not resulted in clinical remission [18]. However, a recent North American phase 3 randomized controlled trial failed to replicate the positive results of earlier studies, leading to the discontinuation of further development and the subsequent withdrawal of this cell-based formulation from European markets, where it had previously been approved [19].

### Clinical trials

Despite a negative phase 3 randomized controlled trial (RCT), there was previous extensive clinical trial and data and real-world evidence demonstrating both safety and efficacy of MSCs for perianal fistulizing Crohn’s disease. Phase 2 and 3 clinical trials with varying trial methodologies (randomized double arm or open label (OL) single arm) data indicate that, in certain cohorts, up to 84% of patients showed high rates of clinical and radiographic healing with minimal adverse events [20]. Meta-analyses of 25 prospective studies investigated the role that local injections of MSCs had on a 596-patient population with perianal fistulizing CD. While only four of these listed studies were RCTs, the rest being OL, this analysis highlights the urgent clinical relevance as 84% of the studies involved patients with refractory perianal disease. Aiming to identify the most effective MSC therapy for perianal fistulizing CD, several various injections were utilized, including adipose derived stem cells (ASCs), bone marrow stem cells (BMSCs), stromal vascular fraction, microfragmented adipose tissue, exosomes from umbilical cord MSCs (UC-MSCs), and UC-MSCs themselves. From this meta-analysis, combined remission (clinical closure plus radiographic conformation: absence of active inflammation and complete closure of the fistula tract) occurred in about 36.2% at 3 months, 57.9% at 6 months, and 52% at 12 months, where optimal efficacy of MSC treatment was achieved at 6 months [21]. In an additional meta-analysis, 43 studies involving 1160 patients sought to determine the efficacy of various stem cell treatments including ASCs and BMSCs. Healing was defined as complete closure of anal fistulas without discharge and epithelization of the external openings, and the results demonstrated healing rates of 63.6% with BMSCs and 60.4% with ASCs [22].

While some of these aforementioned clinical trials utilized MSCs from bone marrow, darvadstrocel (Alofisel), which was the only stem cell therapy that advanced to phase 3 double-blind clinical trials for the treatment of complex perianal Crohn’s disease, was derived from adipose tissue [23]. The initial phase 3 ADMIRE-CD trial demonstrated that a single injection of 120 million allogenic adipose-derived MSCs (darvadstrocel) produced and maintained significantly higher remission rates than placebo without new safety concerns, with 56% of treated patients remaining in clinical remission at 104 weeks versus 40% of patients in control groups [24]. There is also data showing that the durability of this therapy has been confirmed in long-term follow-up studies where over half of patients (56–62%) retain fistula healing up to 4 years and durability of remission extending up to 8 years in some cases [25]. However, in the phase 3 ADMIRE-CD II trial, expanded to a larger global population to validate findings from ADMIRE-CD I, remission rates following the same single allogenic injection in 568 patients (48.8%) did not differ significantly from placebo (46.3%) [26]. The high remission rates among the placebo group in both ADMIRE-CD trials could have been due to the surgical intervention prior to darvadstrocel administration, which involved closing the fistula internal openings with sutures [27].

### Clinical relevance of findings

On the basis of several clinical trials, MSCs were seen to offer a reproducible, durable, and biologically rational regenerative approach to refractory PFCD, delivering 50–80% healing rates, sustained benefit beyond 2 years, and no observed excessive serious adverse events [18, 18]. MSC therapy demonstrated significant efficacy in short-term and long-term sustained healing. While MSCs are unlikely to become a commercially available product following ADMIRE-CD II, the results have established a benchmark for future regenerative injectable products to be used in combination with medical and surgical intervention.

## Platelet-rich plasma in perianal fistulas

### Background

Platelet-rich plasma (PRP) is an autologous blood product with high concentrations of platelets and growth factors, prepared by centrifuging a patient’s own blood, and is used widely in several regenerative medical therapies [28]. In clinical settings, PRP therapy has been applied across many fields including healing in tendinopathy and osteoarthritis in orthopedic settings, skin rejuvenation and scar remodeling in dermatologic care, and ovarian rejuvenation and endometrial repair within gynecology medicine [29]. PRP therapy has gained increasing attention as a biologic adjunct for the treatment of PFCD and serves as a minimally invasive intervention of promoting fistula healing through localized delivery of growth factors and cytokines that drive epithelization, angiogenesis, and macrophage-mediated repair [30]. This biologic rationale parallels MSC therapy, but PRP’s technical simplicity, significantly lower cost, and favorable safety profile make it an appealing complement in regenerative protocols.

### Clinical trials

Evidence from early clinical trials demonstrated moderate yet clinically meaningful healing rates with PRP alone. A phase 2 single-center clinical study of 29 patients with confirmed perianal fistulizing CD given autologous platelet-rich and platelet-poor plasma (PPP) injections reported findings of complete healing in 33% of patients at 24 weeks and 40% at 48 weeks; an additional 38–40% of patients achieved a partial response throughout the duration of the study. Overall, PRP resulted in 71% of patients being asymptomatic at 6 months and 80% at 1 year. The safety profile was somewhat advantageous, with minor local adverse events such as transient pain, but also five adverse events requiring drainage of abscess formation [30].

Another phase II prospective, nonrandomized pilot study investigated combining stromal vascular fraction (SVF) and PRP injection among 25 patients with PFCD. The study found that 37.5% achieved complete radiologic healing in 3 months, 68% had clinical response at 12 months, and 40% achieved complete clinical closure at 12 months. Importantly, there were no major adverse events and both the lipo-harvesting and injection sites healed well without complication. These results underscore the feasibility, safety, and regenerative combination of PRP when used with adipose-derived cellular fractions to improve tissue remodeling and closure of fistula tracts [31]. These findings were further underscored in a 2024 systematic review and meta-analysis of 138 patients compiled from 10 studies that described an overall healing rate of 82.4% and complete healing in 48.1% of cases at 12 months. Subgroup analyses revealed that PRP monotherapy achieved 38.5% complete healing, while PRP combined with SVF showed improved efficacy with 58.62% of patients demonstrating complete healing. Even greater, PRP plus adipose-derived stem cells demonstrated higher efficacy as complete healing was achieved in 85.9% of patients. In addition to efficacy, safety was well established with adverse events occurring in only 5.6% of all cases without a single serious complication reported. This meta-analysis confirmed that PRP-based therapies, particularly when paired with adipose-derived stem cells, can achieve durable remission similar to that observed with MSC injections alone [32].

### Clinical relevance of findings

When considering the data with injection of biologic therapies, cross-study analysis suggests that MSCs were more effective than PRP alone with improved clinical remission rates of 52–83% at 6–12 months compared with 33.3% at 24 weeks and 40% at 48 weeks with PRP alone [30]. However, owing to varied approaches and techniques utilized in included studies, concrete conclusions are difficult to define, but multimodal therapies have demonstrated optimal outcomes with complete healing of fistulas between 85.9% to 88% [32, 33]. Additionally, PRP may be more accessible and economically feasible than cell therapies in the majority of patients with PFCD.

Regardless of the biologic, across all studies to date, both PRP and cell therapy have been well tolerated without significant adverse events. Reported adverse events have been limited to transient local pain or minor abscesses with no impairment in fecal continence or systemic complications. PRP’s autologous nature eliminates immunogenic risk and logistical barriers associated with allogeneic MSC sourcing. PRP offers a safe, biologically active, and cost-effective adjunct for refractory PFCD. Monotherapy yields 33–40% complete closure within 1 year, whereas combination approaches such as PRP and SVF can achieve up to 68% overall response and 40% radiologic healing in 12 months. Compared with MSC injections, PRP remains less potent but more accessible and can enhance outcomes when paired with conventional medical and surgical approaches. Currently, MSC research is more advanced, with a greater understanding of tissue regeneration and latent potential benefits, which may introduce bias and lead to more favorable outcomes for MSC therapy; therefore, further research is necessary to allow for more objective comparisons. These findings underscore the notion that continuing to investigate regenerative biologic injectables to complement conventional therapy may be beneficial.

## Autologous fat injections in perianal fistulas

### Background

Autologous fat injections are another form of investigational therapy for the improvement of PFCD symptoms. These injections consist of local administration of a patient’s own adipose tissue, harvested via an extraction (i.e., liposuction), which are then injected into or around the fistula tract. This rationale was developed with goals to leverage the regenerative, anti-inflammatory, and immunomodulatory properties of adipose-derived MSCs in promoting fistula healing [34]. As opposed to other MSC-derived therapies that involve harvesting a primary tissue source with subsequent isolation and expansion of MSCs, an often expensive, resource limited, and logistic constrained process, autologous adipose tissue injections may also offer a reasonable treatment approach with less required infrastructure and cost [3][3]. The clinical applications of autologous fat injections are currently being studied and have demonstrated promising results in scarring, fibrosis, atrophy, neuropathic pain, and autoimmune diseases including scleroderma and morphea [36, 37].

### Clinical trials

This technique has been trialed in CD-related studies as well as in complex cryptoglandular perianal fistulizing disease. Protocols usually involve harvesting fat from the abdomen or thigh, minimal processing, and injection into the fistula tract [38]. In a systematic review and single-arm meta-analysis that included 49 patients across four prospective intervention trials, treatment with autologous fat injection resulted in fistula closure of 74% (95% confidence interval (CI) 57–85%). Notably, clinical healing was observed in 20% of patients at 3 months, increasing to 60% at 12 months. These rates are more successful than those previously reported for infliximab, which typically achieve sustained healing in about 36% of cases at 12 months. It was noted that this intervention was well tolerated, with minimal adverse events recorded. Most adverse events, which included proctalgia, abscess formation, pain and bleeding in the fat harvest site, urinary retention, CD flare, and formation of new perianal fistulas, were infrequent and manageable [3].

Additionally, a prospective interventional study included 21 patients with complex PFCD who were injected with autologous adipose tissues. The goals of this study included complete fistula healing, which involved no symptoms of discharge and no visible external or internal fistula openings. At 6 months following the last injection, 57% of patients met the criteria for complete fistula healing. Magnetic resonance imaging (MRI) output demonstrated complete anatomical resolution in 9/10 cases, with the remaining patient exhibiting significant reduction in fistula drainage. Adverse events were reported, including small abscess formation (*n* = 2), transient urinary retention (*n* = 1), and bleeding at the donor site during liposuction (*n* = 1). These results indicate that injection of freshly harvested autologous adipose tissue appears to be a safe and moderately effective therapy for complex PFCD [39].

### Clinical relevance of findings

Clinical findings indicate that autologous fat injections are a safe and promising option for the treatment of PFCD, with healing rates ranging from approximately 57% to 74% at 6–12 months. A prospective study reported complete fistula healing in 57% of patients at 6 months, with MRI confirmation in most cases and minimal adverse events [39]. Another single-arm meta-analysis found pooled clinical healing or closure rates of 74% at 6–12 months, again, with minimal complications [3]. Additionally, when discussing combining approaches, long-term results from a pilot study assessing platelet-rich stroma (PRS), which was prepared using a combination of SVF and PRP, demonstrated positive outcomes, with 88% of patients exhibiting complete clinical closure and 100% of patients exhibiting partial clinical closure at the long term follow-up visits [40]. Follow-up data suggest that autologous fat injections offer a robust and effective therapy for long-term fistula healing in most patients and that repeat treatment also serves as effective for those who experience reopening of the fistula tract [38] (Table 1).

**Table 1:** Novel PCFD therapies

	Benefits	Next steps
Mesenchymal stem cells (MSCs)	Local regenerative therapy with anti-inflammatory, immunomodulatory, and pro-angiogenic effects [15] High clinical and radiographic healing in selected cohorts (up to 84%) with minimal adverse events [20] Meta-analysis remission rates of 36.2% at 3 months, 57.9% at 6 months, 52% at 12 months, peak efficacy at 6 months [21] Durable response with sustained remission beyond 2–4 years; long-term healing maintained in 56–62% of patients [24, 25] Favorable safety profile with no excess serious adverse events reported [20–22]	Standardization of MSC source, dose, and delivery techniques to reduce heterogeneity across trials [20–22] Evaluation of MSCs in combination with surgical closure techniques and concurrent medical therapy [18, 27] Long-term real-world studies to further assess durability, safety, and cost-effectiveness in refractory PFCD [24, 25]
Platelet-rich plasma (PRP)	Autologous, minimally invasive biologic therapy delivering concentrated growth factors to promote epithelization, angiogenesis, and tissue repair [28, 30] Favorable safety profile with low immunogenic risk and minimal logistical barriers compared with cell-based therapies [30] Meta-analysis outcomes: 82.4% overall healing, 48.1% complete healing at 12 months across PRP-based therapies [32] Cost-effective and widely accessible regenerative therapy compared with MSC-based therapies [30, 32]	Standardization of PRP preparation methods, platelet concentration, and injection protocols to improve reproducibility [30, 32] Larger randomized controlled trials to better define PRP efficacy relative to MSCs and conventional surgical approaches [30, 32] Further evaluation of PRP as part of multimodal regenerative strategies, particularly in combination with SVF or adipose-derived stem cells [31–33] Long-term follow-up studies to assess durability of fistula closure and sustained symptom remission [30, 32]
Autologous fat injections	Autologous regenerative therapy leveraging anti-inflammatory and immunomodulatory effects of adipose-derived MSCs [34] Lower cost and reduced logistical complexity compared with isolated and expanded MSC-based therapies [3, 35] Complete healing in prospective studies: 57% complete fistula healing at 6 months with MRI-confirmed resolution in most cases [39] Favorable safety profile with predominantly mild and manageable adverse events related to harvest or injection sites [3, 40]	Larger randomized controlled trials to confirm efficacy and compare outcomes with MSC- and PRP-based therapies [3, 39] Standardization of fat harvesting, processing, and injection techniques to improve reproducibility across centers [38] Further investigation of combination strategies incorporating PRP or SVF to enhance regenerative efficacy [40] Long-term follow-up studies to better define durability of healing and need for repeat interventions [38, 40]

## Limitations of the above technologies

The use of MSCs, PRP, and autologous fat injections for perianal fistulizing Crohn’s disease represents a promising advance, but each modality is limited by important challenges in clinical application.

Mesenchymal stem cells (MSCs) have demonstrated safety and moderate efficacy in refractory perianal Crohn’s disease, with healing rates ranging from 50% to 83% at 6–12 months in phase II and III trials. However, limitations include variable protocols (autologous versus allogeneic sources, bone marrow versus adipose tissue, cell dose, and use of scaffolds), lack of standardization, comparison of results from single-arm and meta-analyses with variable methodologies, and restricted availability outside specialized centers. Long-term durability remains uncertain, with some studies reporting recurrence or need for re-intervention beyond 1 year. Adverse events are infrequent but include anorectal pain and abscess formation. Cost, regulatory hurdles, pharmaceutical buy-in after darvadstrocel’s lack of performance, and the need for specialized expertise further limit widespread adoption [14, 16, 31–33].

Platelet-rich plasma (PRP) is well tolerated and may enhance healing, especially when combined with stromal vascular fraction or stem cells. However, PRP alone yields lower complete healing rates (38–48%) compared with combination approaches, and its efficacy is highly variable across studies. There is a lack of large, randomized controlled trials, and optimal preparation, dosing, and injection protocols are not standardized. Additionally, the conclusions drawn were primarily from meta-analyses with limited consistency among methodologies. Minor adverse effects such as localized pain are reported, but long-term safety and durability data are limited [30–33, 41].

Autologous fat injections (including microfragmented adipose tissue and stromal vascular fraction) have shown feasibility and safety, with pooled clinical healing rates of 57–74% at 6–12 months and up to 70% at 3 years in small studies. The need for lipo-harvesting, variable definitions of healing, and lack of controlled comparative data restrict generalizability. As mentioned previously, these results are derived from single-arm trials and meta-analyses with moderate heterogeneity and limited sample sizes. Adverse events are rare but include post-procedure pain, abscess, and minor bleeding. The long-term durability and optimal patient selection remain to be defined, and ongoing randomized trials are needed to clarify the role of fat grafting in the therapeutic arsenal [3, 38, 39, 42].

### Common adverse event: abscess formation

Perianal abscess formation is a common complication across the three needle-based interventions for perianal fistulas discussed above. Abscesses may develop owing to premature tract closure, local tissue reaction to injected materials, or procedural trauma. While this is an established adverse event among therapies for PFCD, the rates vary among the different interventions. Patient selection and post-procedural monitoring remain critical in prevention of perianal abscess formation. Exclusion of patients experiencing active abscesses prior to initiation of needle-based therapies and ensuring all abscesses have been drained should be prioritized to optimize patient recovery and long-term healing. Additionally, rigorous surgical preparation could contribute to decreased abscess formation, including thorough curettage, closure of internal openings, and a controlled injection technique [43].

Across all three technologies, limitations include the absence of standardized endpoints, heterogeneity in patient selection and procedural technique, and insufficient long-term data. These factors complicate direct comparison with established medical and surgical therapies and highlight the need for further research to optimize and position regenerative therapies in PFCD management.

## Future directions

Despite the limitations of current regenerative therapies for PFCD, the field is rapidly evolving with promising innovations that address existing challenges and offer new therapeutic paradigms. Future directions encompass optimization of existing cell-based therapies, development of cell-free alternatives, integration of novel adjunctive techniques, refinement of classification systems, and rigorous evaluation of cost-effectiveness and long-term outcomes.

### Optimization and standardization of regenerative therapies

A critical priority is the standardization of protocols for MSC therapy, PRP, and autologous fat injections. Current heterogeneity in cell source (autologous versus allogeneic, bone marrow versus adipose tissue), preparation methods, cell dose, use of scaffolds, and injection techniques limits the ability to compare outcomes across studies and implement best practices. Future research should focus on identifying optimal cell doses, timing of administration, and patient selection criteria through well-designed randomized controlled trials [15, 18]. The development of standardized endpoints, including validated definitions of clinical, radiological, and combined remission, will facilitate meaningful comparisons and meta-analyses [5, 18].

### Combination approaches

Emerging evidence suggests that combining regenerative therapies may enhance efficacy beyond single-modality treatment. The combination of SVF with PRP has demonstrated complete healing rates of 58–88% at long-term follow-up, significantly higher than PRP alone. Similarly, the addition of microfat to stromal vascular fraction cells has shown sustained healing in 70% of patients at 3 years. Additionally, evidence suggests that combination approaches, including the use of antibiotics, may be more effective. Patients treated with adalimumab in combination with the antibiotic ciprofloxacin demonstrated a 71% clinical response rate and 65% remission rate compared with those receiving adalimumab monotherapy, who showed a 47% clinical response rate and a 33% remission rate [44].

A prospective single-arm study aiming to establish the efficacy and safety of autologous blood clot product as a potential therapeutic for complex perianal fistulas and PFCD reported 69% complete healing at 6 months and 71% clinical remission at 1 year, respectively [45]. Future trials should systematically evaluate combination regimens, including the integration of regenerative therapies with advanced surgical techniques such as fistula curettage, internal opening closure, and endorectal advancement flaps. The synergistic effects of combining cell-based therapies with optimized medical management, particularly anti-TNF agents and other biologics, warrant further investigation to maximize healing rates and durability [5, 30–33, 41, 42, 46].

### Clinical injection techniques and fistula tract preparation

**Fig. 1:**
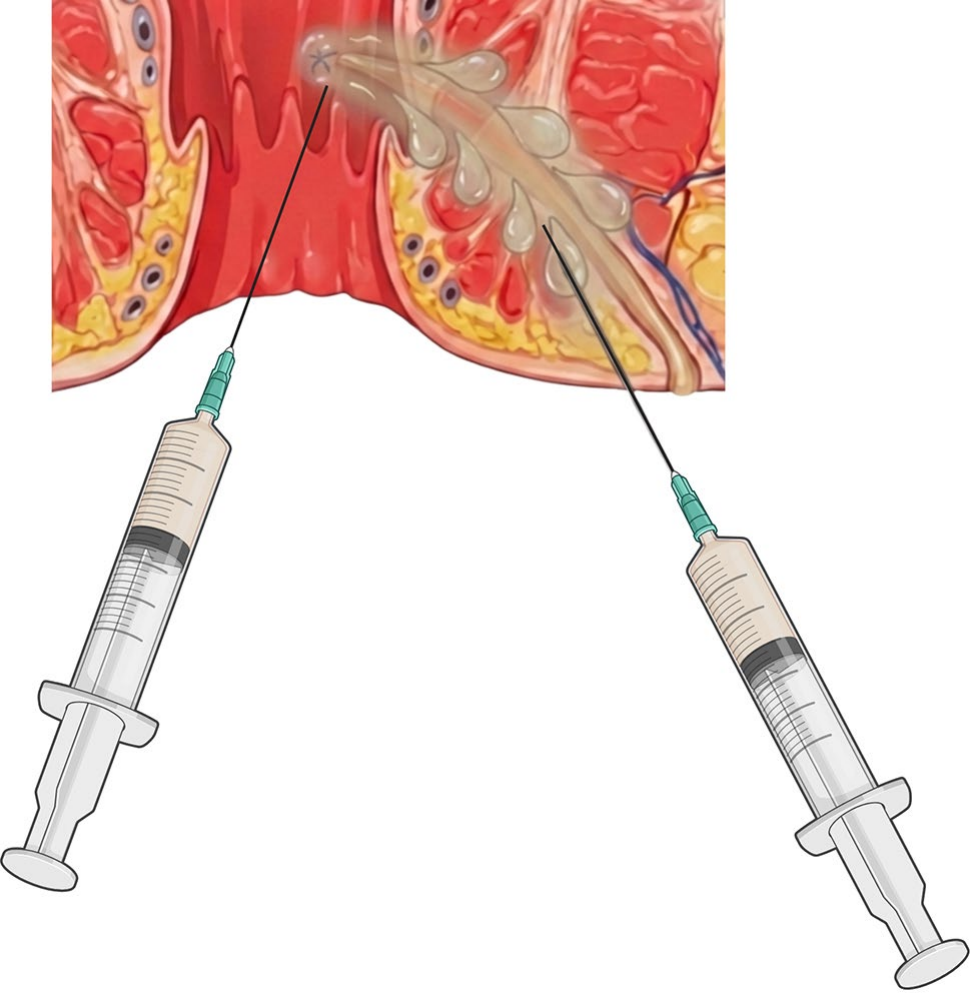
Clinical injection technique for perianal fistulas [48]

Physicians employ a wide range of approaches when managing perianal fistulas prior to attempting the latter novel therapies to prepare the tract for injection. Seton placement is a common procedure that attempts to maximally drain the fistula of fluid to better achieve healing and to reduce the risk of abscess formation. Seton procedures usually entail surgically suturing the fistula tract from the internal to the external opening [47]. Additionally, fistula curettage is another practice implemented to optimize fistula closing where fibrous and infected tissue lining is removed. Injection techniques involved injecting the surrounding tissue about 2 mm from the internal opening curettage site and extending the injections into all quadrants of the fistula tract [31] (Fig. 1).

### Extracellular vesicle-based therapies

MSC-derived extracellular vesicles (EVs) represent a promising cell-free alternative that may overcome many limitations of traditional cell therapy. EVs mediate the therapeutic effects of MSCs through paracrine signaling, delivering anti-inflammatory, pro-angiogenic, and immunomodulatory factors without the need for viable cell engraftment. Preclinical studies demonstrate that MSC-derived EVs suppress inflammatory cytokine production, promote wound healing, and achieve fistula closure in animal models of PFCD. EVs offer several advantages over whole-cell therapy, including easier storage and transport, reduced immunogenicity, lower risk of tumorigenicity, and potential for off-the-shelf availability. Clinical trials evaluating EV therapy for perianal fistulizing Crohn’s disease are needed to establish safety, optimal dosing, and efficacy compared with MSCs [12, 15, 49, 50]. Additionally, strategies to enhance EV production and potency, such as upregulation of autophagy in MSCs, may further improve therapeutic outcomes [49, 50].

### Novel adjunctive therapies

Adjunctive therapies that enhance the local wound healing environment represent another promising avenue. Hyperbaric oxygen therapy (HBOT) has been investigated as a means to improve tissue oxygenation and promote fistula healing, though robust clinical trial data are still needed. Exclusive enteral nutrition and other dietary interventions may reduce luminal inflammation and support fistula closure, particularly when combined with surgical and regenerative approaches. Fistula conditioning techniques, including curettage and debridement, may optimize the fistula tract for subsequent cell-based therapy by removing epithelialized tissue and promoting a pro-healing microenvironment. Future research should evaluate these adjunctive strategies in controlled trials to define their role in the therapeutic algorithm [12, 31, 33].

### Advanced classification and personalized care

The adoption of comprehensive classification systems such as TOpClass will facilitate personalized treatment planning and improve standardization in clinical practice and research. TOpClass incorporates clinical and anatomic characteristics to assess disease severity, guide treatment selection, and align interventions with patient goals. This framework enables tailored application of regenerative therapies based on fistula complexity, prior treatment history, and individual patient factors [5]. Future studies should leverage TOpClass and similar systems to stratify patients in clinical trials, identify predictors of response to regenerative therapies, and develop evidence-based treatment algorithms.

### Cost-effectiveness and health economic analyses

As regenerative therapies become more widely available, rigorous cost-effectiveness analysis will be essential to inform clinical decision-making and healthcare policy. Preliminary data suggest that MSC therapy is more cost-effective than fecal diversion for refractory PFCD, with lower overall costs and improved quality-adjusted life years. Future studies should evaluate the cost-effectiveness of PRP, autologous fat injections, and combination therapies compared with conventional medical and surgical approaches, incorporating long-term outcomes, recurrence rates, and quality of life measures [51]. Such analyses will be critical for securing reimbursement and ensuring equitable access to these innovative treatments.

### Long-term durability and recurrence prevention

A major knowledge gap is the long-term durability of healing achieved with regenerative therapies. While short- to medium-term outcomes are encouraging, data beyond 3 years are limited. Future research should include extended follow-up periods to assess recurrence rates, identify factors associated with sustained remission, and determine the need for repeat interventions [18, 41, 42]. Understanding the mechanisms underlying fistula recurrence including persistent inflammation, incomplete tract obliteration, and ongoing immune dysregulation will inform strategies to prevent relapses and optimize long-term outcomes.

## Multicenter randomized controlled trials

The current evidence base for regenerative therapies in PFCD is derived largely from single-center studies, pilot trials, and meta-analyses with moderate heterogeneity. Large, multicenter, randomized controlled trials are urgently needed to definitively establish the efficacy, safety, and optimal use of MSCs, PRP, and autologous fat injections [3, 32]. Ongoing trials, such as those evaluating stromal vascular fraction with PRP (NCT04010526), will provide critical data to guide clinical practice. Future trials should incorporate standardized endpoints, rigorous radiological assessment, patient-reported outcomes, and long-term follow-up to comprehensively evaluate these therapies [42].

In summary, the future of regenerative medicine for PFCD is filled with opportunities to optimize existing therapies, develop novel cell-free approaches, integrate adjunctive techniques, and personalize care through advanced classification systems. Continued innovation, rigorous clinical investigation, and multidisciplinary collaboration will be essential to translate these advances into improved outcomes for patients with this challenging disease.

## Data Availability

No datasets were generated or analyzed during the current study.
